# Exploring the regulatory mechanism of osteoporosis based on intestinal flora: A review

**DOI:** 10.1097/MD.0000000000032499

**Published:** 2022-12-30

**Authors:** Kasimu Awuti, Xukai Wang, Liquan Sha, Xiangyang Leng

**Affiliations:** a College of Traditional Chinese Medicine, Changchun University of Chinese Medicine, Changchun, Jilin Province, China; b The Affiliated Hospital of Changchun University of Chinese Medicine, Changchun, China.

**Keywords:** bone metabolism, intestinal microflora, osteoporosis, postmenopausal osteoporosis

## Abstract

Osteoporosis is 1 of the common diseases of bone metabolism in clinic. With the aging of the population in China, osteoporosis is becoming more and more serious, and it has become 1 of the major public health problems. However, traditional therapies, such as calcium therapy and estrogen therapy, can cause serious adverse effects and damage to the body when ingested over a long period of time. Therefore, there is an urgent need to explore alternative therapies with less side effects in clinical practice. Intestinal flora is a hot topic of research in recent years. It has been studied in inflammatory bowel disease, diabetes, depression and so on. Recently, intestinal flora has received increasing attention in the pathways regulating bone metabolism. This paper contains a review of recent studies related to osteoporosis and gut flora in terms of its metabolites, immune, endocrine, and brain-gut axis pathways. The strong association between intestinal flora and bone metabolism suggests, to some extent, that intestinal flora can be a potential target for osteoporosis prevention and treatment, providing new ideas and therapies for the prevention and treatment of osteoporosis.

## 1. Introduction

There are trillions of microbes in the human body, including bacteria, archaea, viruses, fungi and other forms of microbes. The unique genes encoded by microbial genomes are 100 times more than those of human genomes.^[1,2]^ Different organs have different microflora, but according to the available data, intestinal microflora, as an important part of the human body, is closely related to human health, and has been paid more and more attention to.^[3–6]^ In recent years, with the rapid development of the research on intestinal flora, it is found that the complex ecosystem formed by intestinal flora constantly interacts with its host and environment, resulting in the imbalance of intestinal flora, accompanied by more health problems. This has become a hot spot for researchers to study the relationship between intestinal flora and other diseases, including digestive diseases, diabetes, rheumatic diseases, Parkinson’s disease, cancer, et cetera.^[7–10]^

Osteoporosis is the most common systemic bone disease, which is closely related to heredity, gender, age and postmenopausal status,^[11,12]^ Mainly manifested as bone loss and destruction of bone structure. It is estimated that in 2010, the number of people aged 50 and above who are at high risk of fracture in the world was 158 million. By 2010, this number may double.^[13,14]^ According to a national survey report for China, the prevalence rate of osteoporosis in people over 50 years old in China is 19.2%. In addition, the prevalence rate of osteoporosis in men is 6.0%, that in women is as high as 32.1%, and that in women over 65 is as high as 51.6%, At the same time, the prevalence of osteoporosis is different in different areas of China.^[15–17]^Because osteoporosis has no obvious clinical symptoms before the first osteoporotic fracture, it is also called asymptomatic disease. If osteoporosis is not found and treated early, once osteoporotic fracture occurs, it will require a lot of manpower, material resources and financial resources, resulting in heavy family and social burden. Therefore, it has become 1 of the major public health problems in China and the world.^[18,19]^ In an environment of surging prevalence, it has become a top priority to address this heavy burden.

With the in-depth study of intestinal flora, some orthopedic researchers have turned their attention to intestinal flora and orthopedic diseases. Many studies have confirmed the close relationship between intestinal flora and musculoskeletal health and diseases,^[20–23]^ Tousen et al^[24]^ By giving isoflavones and fructooligosaccharides that can change intestinal flora to osteoporotic Wistar rats after ovariectomy, after 90 days of culture, it is found that compared with sham operation group, the total mineral density of femur and metaphysical bones of Wistar rats is significantly increased, and the bone strength is also significantly improved, suggesting that there is a close relationship between intestinal flora and osteoporosis. Therefore, this paper expounds the immune mechanism, endocrine mechanism, brain-gut axis mechanism, the effect of metabolites on intestinal flora and the regulatory mechanism of osteoporosis. The aim is to provide a new alternative therapy for clinical prevention of osteoporosis. As well as providing readers with strong theoretical support and data support for research in related fields.

### 1.1. Intestinal microorganisms regulate osteoporosis through their metabolites

Compared with adults, the intestinal flora of human beings is simple and limited at birth. These flora structures have changed greatly with birth mode, infant feeding, life style, drug treatment, host inheritance, et cetera. The diversity of intestinal flora structure will converge to that of adults at the age of 3, so intestinal flora in infancy is highly plastic.^[25–27]^ Intestinal flora plays an important role in human metabolic diseases.^[28–30]^ Studies show that the operation of microbial communities or their metabolites may provide the ability to optimize bone growth and health.^[31]^ Osteoporosis is essentially due to abnormal bone metabolism, that is, the balance maintained by osteoblasts and osteoclasts is broken.^[32]^So how exactly do intestinal flora play a role in regulating osteoporosis through their metabolites? This brings us to bile acids and bacteriophages. Bile acids are an important signal molecule of bile components and synthesis and secretion of liver cells. About 95% of bile acids are returned to the liver through “enterohepatic circulation” and secreted into bile. Intestinal flora transforms bile acids bound with amino acids into unbound bile acids in the liver, so intestinal flora plays an important role in bile acid metabolism.^[33]^ Some bile acids have adverse effects on primary human osteoblasts and osteosarcoma cells, which can reduce bone differentiation and mineralization, and increase apoptosis.^[34]^ Short-chain fatty acids (SCFAs) are microbial end products fermented by intestinal bacteria. Short-chain fatty acids are organic fatty acids containing < 6 carbon atoms, including acetic acid, propionic acid, butyric acid, isobutyric acid, valeric acid and isovaleric acid.^[35]^ SCFAs is mainly metabolized by Bacteroides, Lactobacteriaceae and a variety of negative bacteria in intestinal flora, which has a strong ability to regulate bone metabolism.^[36]^Butyrate ensures that metabolites produced by healthy microbial communities endow epithelial barrier integrity and immune homeostasis, and inhibits osteoclast differentiation by destroying nuclear factor kappa-B nuclear translocation induced by tumor necrosis factor-*α* (TNF-*α*) and activating p 38 MAPK signal pathway mediated by nuclear factor-κ B receptor activator.^[37,38]^ The binding products of propionate and butyrate are expressed by immature osteoclast precursors, which can inhibit osteoclast differentiation when reaching appropriate doses.^[39]^In addition, SCFAs may stimulate the formation of osteoblasts through insulin-like growth factor I (IGF-1)-mediated bone net anabolic metabolism.^[40]^SCFA participates in SCFA-mediated osteoclast development inhibition through G-protein coupled receptor and free fatty acid G-protein coupled receptor.^[41]^ SCFAs also play an important role in bone formation and mineralization by regulating osteoprotegerin and Wnt signal pathway.^[42]^

### 1.2. Intestinal microbes regulate osteoporosis through immune mechanisms

Autoimmune changes caused by the imbalance of intestinal flora can lead to the loss of bone minerals and the decrease of bone formation, which has been confirmed and expounded by scholars.^[43,44]^ The ecological imbalance of intestinal flora will increase the abundance of bacteria, which can degrade mucin and inhibit the production of mucin. The decrease of mucin will activate nuclear factor kappa-B pathway, and increase TNF-*α*, interleukin-1, Interferon gamma and other pro-inflammatory cytokines in epithelial cells. The elevation of Interferon gamma level will rearrange actin cytoskeleton. When the integrity of epithelial cells is destroyed, intestinal permeability will increase, and the concentration of osteoporosis-related metabolites in blood circulation will increase, resulting in a large number of osteoclast-producing factors such as TNF-*α*. TNF-*α* will lead to the production of receptor activator of NF-kB ligand and macrophage colony-stimulating factor, and at the same time down-regulate osteoprotegerin, thus promoting the activation of osteoclasts and bone resorption.^[29,45]^ Xie et al^[29]^ Experiments show that Qinge Pill can affect bone metabolism by restoring intestinal flora structure, that is, Qinge Pill can regulate intestinal flora structure, inflammatory factor and SCFAs content in intestinal environment of ovariectomized rats, and it can also affect the special sequence of type B collagen related to bone metabolism (special sequence of β-collagen, N- terminal calpain and N-terminal propeptide of total procollagen, etc.).Qing ‘e Pill also affects the expression of vitamin D receptor mRNA in ovariectomized rats, so as to effectively controlling osteoporosis.^[46]^

### 1.3. Intestinal microbes regulate osteoporosis through endocrine mechanisms

It is well known that endocrinology is closely related to metabolic diseases. These include diabetes mellitus, gout,^[47]^ obesity,^[48]^ gout, et cetera. But can endocrine mechanisms regulate osteoporosis? The results suggest that endocrine mechanism play an important role in regulating osteoporosis,^[49]^ The main substances involved in regulation include estrogen, parathyroid hormone, insulin growth factor -1 and serotonin. Estrogen deficiency can cause intestinal flora change, intestinal epithelial barrier function change and immune system dysfunction, which may promote harmful metabolites of intestinal pathogenic bacteria to enter the human body through intestinal epithelial barrier, and then promote immune cells to produce osteoclast promoting factors, thus promoting osteoclast activation and bone absorption.^[50]^ Intestinal flora affects bone mineral density and regeneration rate by regulating the interaction between osteoblasts and immune cells, and has a positive effect on the maturation of host immune cells.^[51,52]^ The decline of estrogen level in postmenopausal women is an important factor of osteoporosis. As a nonsteroidal estrogen analog, S- estrogen has been found to improve related parameters of bone metabolism in osteoporosis.^[53]^ Parathyroid hormone (PTH) is a kind of bone regulation hormone, which needs intestinal flora to play its role in bone catabolism and bone anabolism. PTH works by binding to PTH- parathyroid hormone-related protein receptor, which is expressed in stromal cells, osteoblasts, bone cells and T cells. One of the key mechanism of PTH-induced bone resorption is inducing bone cells to produce RANK, thus increasing the number of osteoblasts and promoting bone formation.^[54]^ IGF-1 can increase bone mass of patients with osteoporosis and promote bone synthesis.^[55,56]^ IGF-1 is mainly synthesized by liver cells in a growth hormone-dependent way, and also produced by bones and muscles. IGF-1 is also involved in bone remodeling and improves bone activity. IGF-1 also participates in the proliferation and differentiation of osteoclasts through mitogen, and regulates osteocytes through endocrine and paracrine mechanisms to promote bone formation.^[57,58]^ Studies show that intestinal flora affects bone metabolism through IGF. The weight and body length of weaned normal Nottobel young mice and infertile Nottobel young mice are the same, indicating that the nutritional status is the same at this time. However, after 2 months of feeding in different nutritional states (sterile feeding and conventional feeding), compared with conventional mice, sterile mice lost 14.5% of their body weight and shortened 4% of their body length. All growth parameters, including femoral length, cortical thickness, cortical bone fraction and trabecular bone, were lower than those of conventional mice. Moreover, the levels of circulating IGF-1 and IGF-BP-3 (itsmain binding protein) in conventional mice were higher than those in sterile mice, which further explained the importance of intestinal flora influencing bone growth through IGF-1.^[59]^

Serotonin is 5- hydroxytryptamine. Studies have shown that serotonin has an inhibitory effect on bone formation, and that the compounds LDL receptor-associated protein 5 and LP533401 promote bone formation by impairing serotonin. Serotonin produced under the control of Tph2 is expressed on brainstem neurons via serotonin 2C signaling on ventral medial hypothalamic neurons and cyclic AMP effector element binding proteins, resulting in reduced sympathetic output, which leads to increased bone formation and reduced bone resorption.^[60]^ It was found that the concentration of 5- hydroxytryptamine conventional colonized mice was significantly higher than that in aseptic mice, and orogenic bacteria in intestinal flora regulated 5 - HT in serum intestinal tract and feces, while the concentration of 5 - HT in aseptic mice would recover after transplanting orogenic bacteria.^[61]^

### 1.4. Intestinal flora regulates osteoporosis through the brain-intestine axis

The brain-gut axis is composed of a complex network, involving neuroendocrine and immune signal pathways and bidirectional neural mechanisms. Importantly, changing the intestinal microbiome will change the brain-intestine axis, which may lead to many diseases.^[62,63]^ Intestinal flora and brain-gut axis interact through a variety of mechanisms. On the 1 hand, intestinal flora can produce various active substances (such as scfa, bile acids, amino acids, intestinal hormones, et cetera.), and some scfa can send signals to the brain by entering the circulatory system^[64]^; Microorganisms can also mediate the metabolism of tryptophan, thus regulating the signal of serotonin and affecting the central nervous system in some cases.^[65]^ Intestinal flora will influence the production of pro-inflammatory and anti-inflammatory cytokines, and then intestinal flora sends signals to the brain through the circulatory system. On the other hand, intestinal flora can also respond to the brain-intestine axis. For example, vagus nerve is closely related to the signal transduction of microflora-intestine-brain axis, and is 1 of the key communication modes between intestine and brain.^[66]^ Vagotomy is used for the treatment of peptic ulcer. More and more research has shown that,^[67]^ Intestinal flora can regulate many bone metabolism through intestinal-brain axis, and its imbalance can cause the imbalance of osteoblasts and osteoclasts, which leads to bone mass reduction.

## 2. Summarize and prospect

The mature intestinal flora structure of the human body is generally established at 1 to 3 years old, and then it remains relatively stable. With the continuous growth of the body, changes of living environment, dietary conditions and other factors, the structure of intestinal flora is constantly changing, and its diversity will obviously decrease, which is very obvious in the elderly. Osteoporosis mostly happens in the elderly, especially in postmenopausal elderly women. Therefore, intestinal flora is closely related to osteoporosis. The metabolites of intestinal flora play a key role in the physiological and pathological process of osteoporosis, and are related to the pathogenesis of osteoporosis. They can be used as biomarkers for early diagnosis and prognosis of osteoporosis in the future; The immune system, endocrine system and brain-gut axis system of intestinal flora play an important role in maintaining the homeostasis and the diversity of intestinal flora. Once the organism system is abnormal, the structure and function of intestinal flora will change, which will inevitably affect the occurrence or aggravation of diseases. The specific regulation mechanism of intestinal flora in regulating human bone metabolism needs to be further studied in order to find a more accurate targets point.

## Author contributions

**Conceptualization:** Xukai Wang, Liquan Sha.

**Data curation:** Xukai Wang.

**Formal analysis:** Xukai Wang.

**Funding acquisition:** Xiangyang Leng.

**Methodology:** Kasimu Awuti.

**Writing – original draft:** Kasimu Awuti.

**Writing – review & editing:** Kasimu Awuti.

## Correction

The corresponding author has been changed from Xukai Wang to Liquan Sha.
